# Research on the Construction of Emergency Network Public Opinion Emotional Dictionary Based on Emotional Feature Extraction Algorithm

**DOI:** 10.3389/fpsyg.2022.857769

**Published:** 2022-04-22

**Authors:** Fang Hui

**Affiliations:** Northwestern Polytechnical University, Xi’an, China

**Keywords:** emotional feature extraction, emergencies, internet public opinion, algorithm, emotional dictionary

## Abstract

How to strengthen emergency management and improve the ability to prevent and respond to emergencies is an important part of building a harmonious socialist society. This paper proposes a domain emotion dictionary construction method for network public opinion analysis of public emergencies. Using the advantages of corpus and semantic knowledge base, this paper extracts the seed words based on the large-scale network public opinion corpus and combined with the existing emotion dictionary, trains the word vector through the word2vec model in deep learning, expands the emotion words, and obtains the candidate emotion words according to the semantic similarity calculation, So as to generate a domain emotion dictionary. The accuracy rate of emotion discrimination by the emotion dictionary constructed in this paper is 0.86, the recall rate is 0.92. Through the verification of accuracy and recall rate, the construction method proposed in this paper has good accuracy and reliability. Because of the great differences in different experiences and situations of different groups, there will be great differences in views and perspectives on the same event. The key to prevent the public from blindly following the crowd should be to reach groups close to emotional distance, and targeted prevention and control of public opinion can be conducted according to different characteristics of different groups.

## Introduction

Emergencies refer to natural disasters, accidents, disasters, public health incidents, and social security incidents that suddenly occur, cause, or may cause serious social harm and need to be dealt with by emergency measures (He and Patnaik, 2018). There are also human and non-human infectious diseases, mass unexplained diseases, food safety, and occupational hazards, animal diseases, and other public health incidents that seriously affect public health and life safety (Delaunay et al., 2016). As mankind enters the 21st century, all kinds of unexpected events are overwhelming, and the whole world is still complicated and difficult to predict and control (Belojevic et al., 2018). Social progress and the development of science and technology not only bring more and more benefits to human beings but also require human beings to have the ability to face and cope with increasing emergencies (Wieder, 2018). Internet public opinion refers to the collection of cognition, attitude, emotion, and behavior tendency of some events expressed by the public using the Internet as a communication medium. The network public opinion of emergencies is a summary of network public opinion information for specific emergencies in a specific environment (Ahlem and Khalil, 2020). The Internet is becoming an important window to reflect social conditions and public opinions. More and more people know the information about emergencies through the Internet and express their views and opinions.

Some researchers put forward the theoretical hypothesis of “noise flow” and the hypothesis of “3F” of crisis communication flow (Edens, 2019). Some scholars have conducted in-depth research on the related fields of China’s public crisis management and government responsibility, including major mass incidents, repeated official corruption cases, and vicious production traffic safety accidents (Rybasack-Smith and Lauro, 2019). Some scholars have suggested that to resolve group emergencies, we must implement the priority strategy of social development (Sharf, 2019). Some scholars also believe that in controlling the occurrence of group emergencies, we should adhere to the principle of social justice and coordinate interests (Perko and Martell, 2020). Other scholars believe that it is necessary to strengthen cultural integration to prevent group emergencies (Rizzo et al., 2021). Some researchers believe that group emergencies are a social phenomenon of improper expansion of civil rights awareness, a deformity of modern legal democracy, and a manifestation of the immaturity of legal democracy (Rodrigues et al., 2020).

After the occurrence of public emergencies, the public will publish public opinion texts, such as posts and comments related to the events through social media, forums, and other online platforms. These texts not only contain topic information related to events, but also contain people’s emotional tendencies toward people, events, different viewpoints, and other objects. Emotion analysis and tendentiousness analysis are one of the important technologies in the field of network public opinion monitoring. Especially under the background of today’s great development of the Internet, network public opinion has the characteristics of pluralism, deviation, dynamics, interactivity, and randomness. In the new century, although the research on value has decreased, it has never stopped (Shaeer and Shaeer, 2016). The problem of values is old and always new. In the new media era, the public not only plays the role of information source but also gradually participates in public events and even becomes the main promoter of public events (Cauley et al., 2016). Surrounded by all kinds of possibilities, people’s values will be shaken in the rethinking of new values. How to make these values continue is also a problem that we should pay attention to. Emotion dictionary is the basis of automatic analysis of public emotions, and it can improve the accuracy of text segmentation based on emotion dictionary. Emotional words used by the public can also be identified through the emotional dictionary, and the emotional type and intensity can be further calculated through the context (Kazmierczak et al., 2020). This paper analyzes and discusses the reshaping of group values by emergencies in the new media environment, analyzes the psychological motivation of emergencies based on the emotional feature extraction algorithm, and constructs a domain emotional dictionary for online public opinion analysis.

The main contributions of this paper are as follows:

1. This paper uses large-scale public emergency papers, combined with a general emotion dictionary and deep learning method, identifies the types and intensities of domain emotion words, and constructs a domain emotion dictionary for public opinion analysis of public emergencies.
2. According to the user’s friend relationship and emotional track on social media, this paper uses social network analysis method to integrate information, such as communication network, relationship network, and interest network, and establishes public opinion monitoring system by identifying opinion leaders, so as to block the spread of rumors.

## Psychological Impact of Public Security Emergencies on the Public and Information Dissemination Mechanism

In the new media environment, information release is no longer the privilege of government, organizations, and traditional media. At the beginning of the new year in 2008, a rare snowstorm occurred in southern China. Due to the stagnant traffic in the snow-covered areas, much traditional media information comes from videos posted by netizens on the Internet. There are also traditional media that keep in touch with passengers trapped on the train through mobile QQ and publish chat records to inform the public of the first scene. As a first-hand news source, these near-live materials inform society of the latest events, which are more real and touching without complicated information processing.

**Figure 1:**
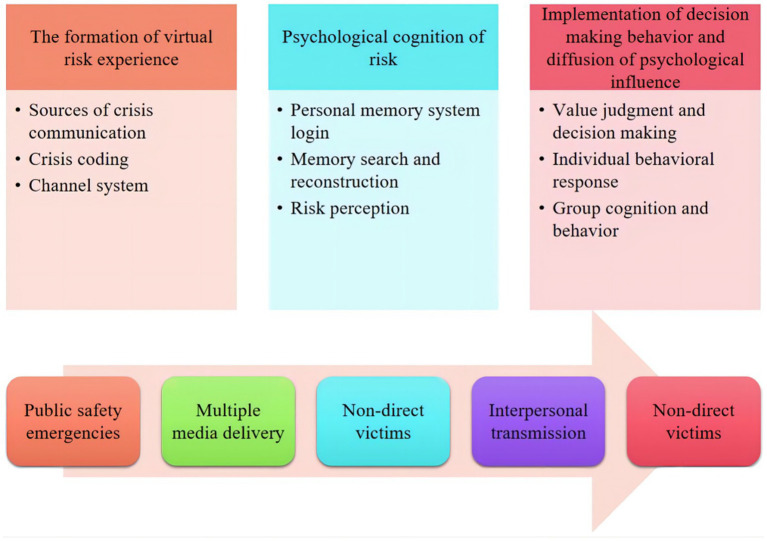
The impact of public safety incidents on public psychology and information dissemination mechanism.

Public security emergencies will not only affect the direct victims and stakeholders, but also the general public. The reason is that with the media’s extensive dissemination of victim information, stakeholders, followers, and the general public will have a virtual risk experience, and then make pressure behaviors. According to the amplification theory of risk society, the psychological influence of the masses can be divided into three stages: the formation of virtual risk experience, the implementation of risk awareness and decision-making behavior, and the diffusion of psychological influence, as shown in Figure 1.

### Psychological Cognition Stage of Risk

People who are not directly affected will judge the probability and influence of similar events around them according to their materials. Implementation of decision-making behavior and diffusion of psychological influence. Due to the role of virtual risk experience, the group receiving event information may have psychological reactions, such as nervousness, panic, and anxiety, and show behaviors, such as searching for information, emergency preparation, and spreading information (Yang and Lin, 2018).

### Social Psychological Causes of Public Security Emergencies

Due to the rapid development of the Internet, the bandwidth or network speed is increasing rapidly, and there are various ways for netizens to express their emotions, such as text information, image information, sound information, and even video information. There are many reasons for public safety emergencies, including political, economic, and social factors. Many scholars focus on external reasons in the research entry point or perspective of the causes of public safety emergencies, and rarely look at and analyze them from the perspective of group members. Therefore, to truly analyze and study public safety emergencies, it is necessary to analyze the psychological characteristics and psychological changes of “groups” and individuals in them (Hassler et al., 2020). From the perspective of social psychology, the author makes a brief analysis of the causes of group psychology and individual psychology in the formation of public safety emergencies.

**Figure 2:**
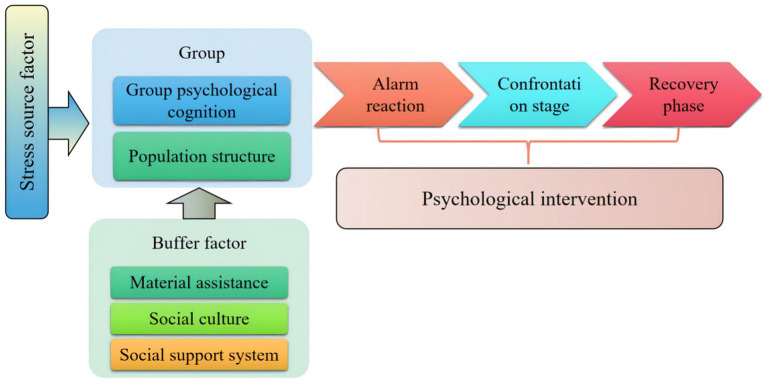
Mechanism of public safety emergencies on group psychological stress response.

Studies have shown that people who have had other traumatic experiences, people who have lost their family life for a long time, poor people, and people who have the pressure of group sex life are more likely to have posttraumatic stress syndrome, so what factors will have an impact on group psychological trauma? The development stage and influencing factor model of group psychological trauma are shown in Figure 2.

### Group Psychological Analysis

For individuals, the material life may return to normal in a short time, while the stress mentality needs to be adjusted and calmed down for a longer time. In the process of psychological intervention for the victim, firstly, it should be clear that the sudden public safety incident has produced a strong and profound painful experience for the individual’s psychology, and the memory often reproduces the traumatic scene. Chinese people’s values always wander between positive “compatibility between wealth and morality” and negative “conflict between wealth and morality” (Siyu, 2020). Experiments have proved that even the simplest “labeling” group classification will lead many individuals to exaggerate the differences between groups and the similarities within groups, thus on the one hand, it may strengthen individuals’ recognition of groups, and on the other hand, it will lead to prejudice against other groups, such as discrimination or hatred. Therefore, the relative deprivation perceived by vulnerable people in psychology may be larger than the actual gap.

**Figure 3:**
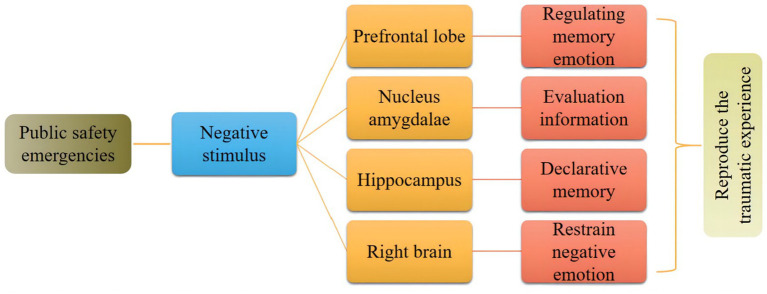
Action mechanism of public safety emergencies on individual psychology.

Psychological trauma caused by public safety emergencies is related to the imbalance of functional activities in different brain regions, pathological cognition, and negative emotions. As shown in Figure 3. The repeated invasion of traumatic psychological disorders may be caused by the inability of some brain structures (such as the amygdala and prefrontal cortex) to effectively inhibit and regulate the forgetting of emotional memory by other brain structures (such as the cortex and hippocampus).

Social psychology research found that people’s self-cognition includes three types of self: individual self, relational self, and collective self, and collective self reflects individuals’ membership in social groups. Through self-classification, a socialized individual always divides himself into multiple groups. The source of network communication is not a professional media audience, and anonymity cannot make the audience more aware of the credibility of the source (Goguen and Gilbert, 2018). At present, there are a large number of anonymous sources in the dissemination of various public security emergencies in China, and there are also a large number of anonymous sources in media reports. Therefore, on the one hand, it may strengthen individuals’ identification with groups; on the other hand, it may lead to prejudice against other groups, such as discrimination or hatred. Therefore, the psychological sense of relative deprivation of vulnerable groups may be greater than the actual gap.

**Figure 4:**
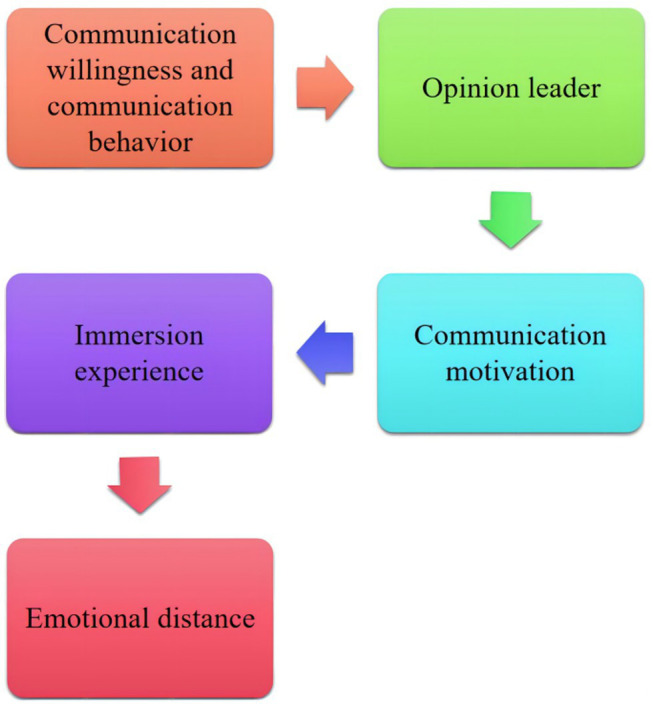
This paper puts forward the flow chart of the hypothesis method.

Intrapersonal communication’s intention is defined as the real idea from the self, which depends on its cognition of information, its knowledge structure, and its values, and is more of a positive and positive communication intention. Whether users are willing to follow the crowd or spread by themselves, it will lead to communication behavior. The hypothetical method proposed in this paper is shown in Figure 4.

From the perspective of emotional distance, this paper discusses the public’s response to public opinion information of public safety emergencies in different emotional distance states. Based on integrating multi-level communication theory, motivation theory, flow theory, and interpretation level, the research model of public opinion communication behavior is constructed. And select “8. 12 Tianjin Big Bang Incident” as an empirical object to study, to explore the law and mechanism of public opinion communication behavior of social media in public emergencies from the perspective of emotional distance, and provide the factual basis for social public opinion management of public safety emergencies.

Therefore, this paper puts forward the following assumptions:

*H1a*: The user’s self-propagation will positively influence the user’s propagation behavior;

*H1b*: Users’ willingness to follow the crowd positively affects users’ communication behavior;

*H2*: Conformity communication intention positively affects self-communication intention.

The more individuals in the community tend to become opinion leaders, the more active they are, and the more sensitive individuals are to the leadership of opinion leaders, the more positive they are to the dissemination of new sources of information. Therefore, this paper puts forward the following assumptions:

*H3a*: Perceived opinion leaders positively influence users’ self-communication willingness;

*H3b*: Perceived opinion leaders positively influence users’ willingness to follow the crowd.

Egoism can be divided into psychological egoism and ethical egoism. Joel Feinberg defines psychological egoism as the ultimate goal that people want or seek its personal interests; ethical egoism means that the individual interests of the actors make an act become a moral act. Self-interest motivation in social public opinion generally includes self-improvement, self-identification, self-status pursuit, and so on. Therefore, this paper puts forward the following assumptions:

*H4a*: Egoism positively affects users’ willingness to self-spread;

*H4b*: Egoism positively affects users’ willingness to follow the crowd.

Emotional distance refers to the emotional difference between the disseminator or audience to the people or things involved in the transmitted content. Generally speaking, the closer the emotional distance is, the more concerned about the event and its state change, and the corresponding degree of attention and communication behavior in social public opinion may also change. Therefore, this paper puts forward the following assumptions:

*H5a*: Emotional distance positively regulates the influence of self-communication intention on communication behavior;

*H5b*: Emotional distance negatively regulates the influence of herd communication intention on communication behavior.

Measurement model analysis is mainly to verify the reliability and validity of variables, while reliability verification is mainly to verify the reliability and consistency of measurement. Validity includes aggregate validity and differential validity. In the part of the reliability test, the Cronbach’s alphas value and combination reliability CR value of each variable are observed, and the results are shown in Table 1.

**Table 1:** Factor load, *α* value, CR, and AVE.

Variable	Measure term	Factor load	Cronbach’s alphas	CR	AVE
Communication behavior	CB 1	0.937	0.934	0.935	0.817
	CB 2	0.921			
Spread the will from the crowd	SWC 1	0.802	0.825	0.893	0.827
	SWC 2	0.889			
	SWC 3	0.831			
Self-propagating willingness	SW 1	0.914	0.873	0.857	0.833
	SW 2	0.893			
	SW 3	0.972			
Altruism	ALT 1	0.833	0.896	0.905	0.815
	ALT 2	0.805			
Self-interest	SLI 1	0.827	0.901	0.882	0.779
	SLI 2	0.893			
Perceptual opinion leader	POL 1	0.885	0.865	0.867	0.814
	POL 2	0.901			
	POL 3	0.874			
Immersion experience	IE 1	0.803	0.882	0.890	0.773
	IE 2	0.877			

It can be seen from Table 1 that the factor load, *α* value and CR value of each variable are all greater than 0.5, which shows that the scale has good reliability.

In the part of validity test, observe the AVE value of each variable and compare the square root of AVE value of each variable with the correlation coefficient value of other variables. The results are shown in Table 2. It can be seen from Table 2 that AVE values of all variables are greater than 0.5, which shows that the scale has good aggregation validity. At the same time, we can see that the square root of AVE value of each variable (the value on the corner line) is greater than the correlation coefficient between this variable and other variables (the value on the non-diagonal line of the corresponding row and column), which shows that the scale has good discrimination validity.

**Table 2:** Square root of AVE value and variable correlation coefficient.

	CB	SWC	SW	ALT	SLI	POL	IE
CB	0.903						
SWC	0.551	0.905					
SW	0.237	0.562	0.884				
ALT	0.339	0.441	0.702	0.836			
SLI	0.452	0.573	0.335	0.645	0.847		
POL	0.284	0.308	0.415	0.428	0.633	0.866	
IE	0.201	0.622	0.208	0.217	0.552	0.327	0.882

In the part of verifying the moderating effect of emotional distance, this paper makes a comparative study by grouping. According to the average value of emotional distance, the sample objects are divided into two groups: near emotional distance group (124 people) and far emotional distance group (206 people), and *T* statistics are constructed based on the analysis data of the two groups to verify the regulatory effect.


(1) $$T=\frac{P_{1}-P_{2}}{\left[\sqrt{\frac{{\left(N_{1}-1\right)}^{2}}{N_{1}+N_{2}-2}S_{1}^{2}+\frac{{\left(N_{2}-1\right)}^{2}}{N_{1}+N_{2}-2}S_{2}^{2}}\right]\left[\frac{1}{N_{1}}+\frac{1}{N_{2}}\right]}$$

In the above formula, *N*_1_ and *N*_2_ represent the sample number of sample 1 (near emotional distance group) and sample 2 (far emotional distance group), respectively; *P*_1_ and *P*_2_, respectively, represent the path coefficients of sample 1 and sample 2 for model test; *S*_1_ and *S*_2_ represent the standard error of model test for sample 1 and sample 2, respectively.

According to the above principles, the verification results of the adjustment effect of emotional distance are obtained, as shown in Table 3.

**Table 3:** Verification of the moderating effect of emotional distance.

Suppose	Close emotional distance		Distant emotional distance		*T* value	
	Path coefficient	Standard error	Path coefficient	Standard error		
H5a: Self-spreading willingness → spreading behavior	0.336	0.087	0.618	0.061	−2.367	Support
H5b: Will spread from the crowd → behavior spread	0.328	0.079	0.032	0.074	2.881	Support

It can be seen from Table 3 that emotional distance positively regulates the influence of self-communication intention on communication behavior, and the *T* value is −2.367, which is significant at the level of *p* < 0.05, assuming that H5a is supported; Emotional distance negatively regulates the influence of herd communication willingness on communication behavior, and the *T* value of 2.881 is significant at the level of *p* < 0.01, assuming that H5b is supported.

**Figure 5:**
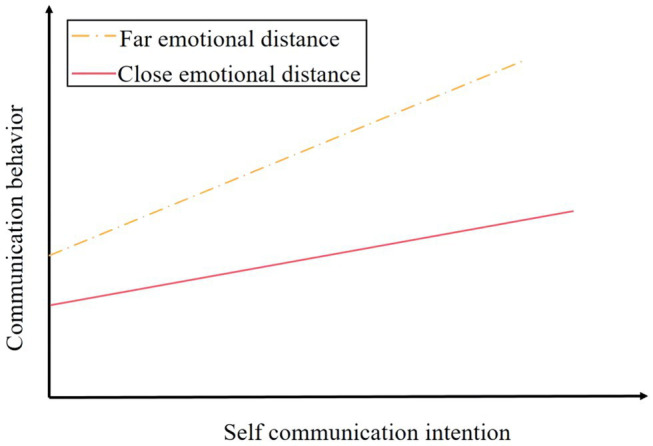
Positive moderating effect of emotional distance.

Assuming that H5a holds, it shows that emotional distance positively regulates the influence of self-communication intention on communication behavior. In grouping and validation of adjustment variables, the path coefficients of the group with near emotional distance and the group with far emotional distance are 0.370 and 0.627, respectively, which are significant at the level of *p* < 0.001. The specific adjustment effect trend is shown in Figure 5.

As can be seen from Figure 4, with the enhancement of self-communication willingness, the growth rate of communication behavior in groups with far emotional distance is relatively fast, while the growth rate of communication behavior in groups with close emotional distance is relatively slow, that is, the farther the emotional distance is, the stronger the real communication behavior transformed from self-communication willingness is.

**Figure 6:**
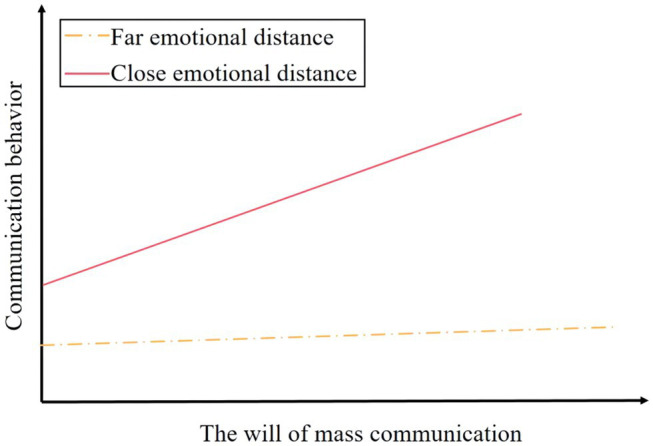
Negative adjustment effect of sensory distance.

Assuming that H5b holds, it shows that emotional distance negatively regulates the influence of herd communication willingness on communication behavior. In the process of grouping validation of adjustment variables, the path coefficient of the group with near emotional distance is 0.361, which is significant at the level of *p* < 0.001, while the path coefficient of the group with far emotional distance is 0.028, which has no significant effect. The specific adjustment effect trend is shown in Figure 6.

It can be seen from Figure 4 that, with the enhancement of herd communication willingness, the communication behavior of groups with close emotional distance grows relatively fast, while the communication behavior of groups with far emotional distance grows very slowly, that is, herd communication behavior tends to occur in groups with close emotional distance, but the possibility of occurring in groups with far emotional distance is relatively small.

## Construction of Emotion Dictionary

**Figure 7:**
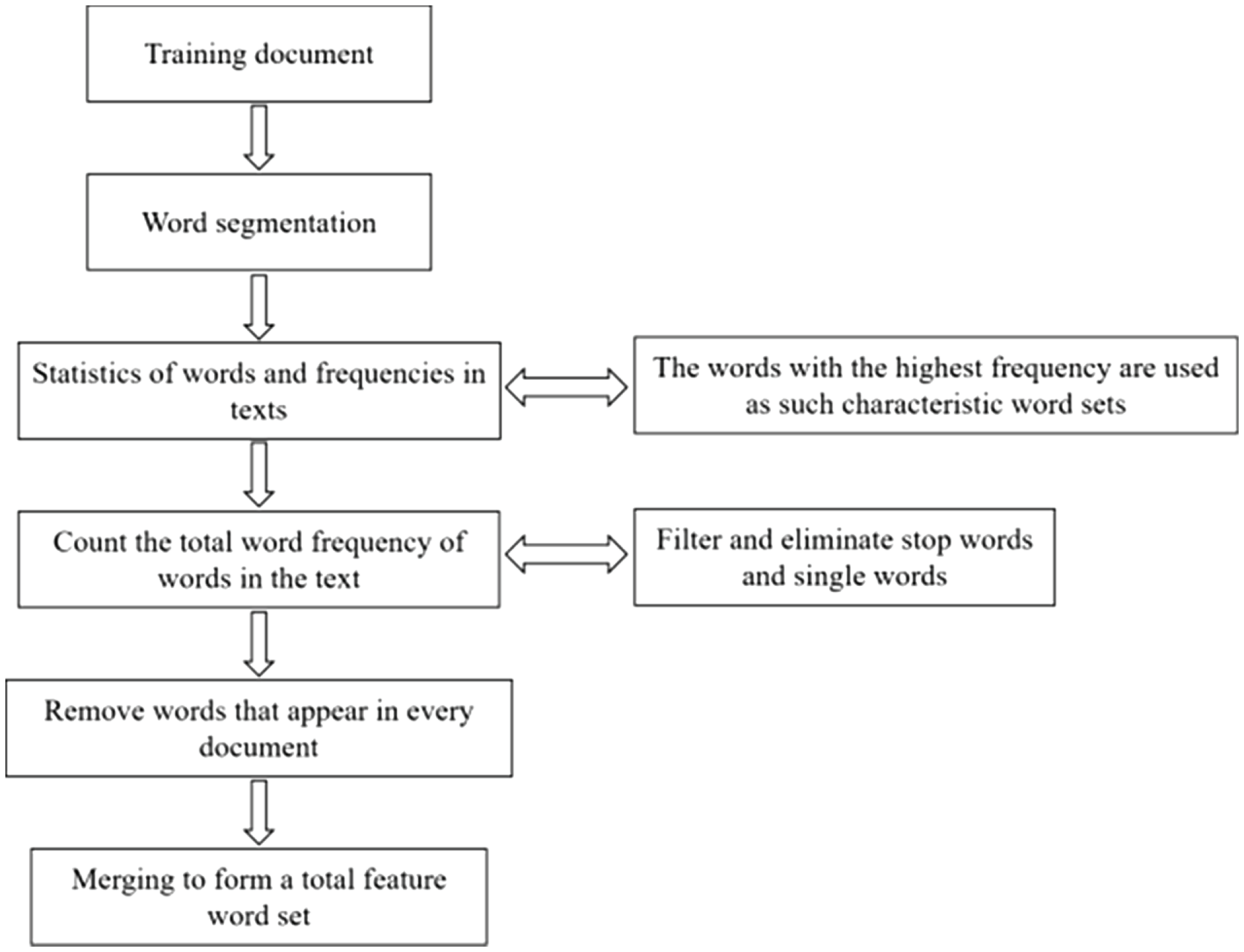
Extraction steps of feature items.

First, the weight is calculated by the word frequency, and then the useless words are eliminated, which are usually irrelevant to the topic. After these words are removed, a new sequence is listed, and then the top four or more words with the highest weight can be selected according to actual needs to represent the core content of the text. The extraction steps of feature items are shown in Figure 7.

Traditional public opinion discussion is usually a one-way influence, that is, after the media releases information, readers can read and analyze it. With the continuous change of Internet technology, online public opinion presents a pattern of interaction among government, media, and netizens. Combined with the reality of fire network public opinion, sentence-level emotional analysis can not only meet the requirements of judging emotional polarity, but also greatly reduce the difficult work, such as attribute clustering and emotion weakening expression processing, which has little effect on the overall accuracy (Trenz, 2016). At present, most of the commonly used emotion dictionaries are general emotion dictionaries, which are lack of specificity when analyzing public emergencies on the Internet. With the emergence of new emotion expressions and emotion words, building a domain emotion dictionary will greatly improve the accuracy of emotion analysis on the Internet. Use training samples to determine the membership function $\mu_{jk}$ of different parameters concerning different emotions. Where *J* is the number of feature parameters, and *C* is the number of emotion categories 1 < j < J, 1 < k < C. $\mu_{jk}$ must meet:


(2) $$\overset{C}{\underset{k=1}{\sum }}\mu_{jk}=1$$

The membership function adopts Gaussian distribution form:


(3) $$\mu_{jk}\left(x\right)\frac{e^{\left[-\frac{1}{2}{\left(\frac{x-m_{jk}}{\sigma_{jk}}\right)}^{2}\right]}}{\overset{C}{\underset{k=1}{\sum }}e^{\left[-\frac{1}{2}{\left(\frac{x-m_{jk}}{\sigma_{jk}}\right)}^{2}\right]}}$$

Here, $m_{jk}$ and $\sigma_{jk}$ , respectively, calculate the mean value and variance of each feature parameter in this type of emotion for different emotions. Using N samples, calculate the average fuzzy entropy of the j-th feature parameter relative to the k-th sentiment:


(4) $$H_{jk}\left(\mu \right)=\frac{1}{N}\overset{N}{\underset{i=1}{\sum }}S\left(\mu_{ik}\left(x^{i}\right)\right)=\left\{\frac{1}{N}\overset{N}{\underset{i=1}{\sum }}\left\{-\mu_{jk}\left(x^{i}\right)\ln\left(\mu_{jk}\left(x^{i}\right)\right)\right\}\ln\left[1-\mu_{jk}\left(x^{i}\right)\right]\right\}$$

The larger the value of the average fuzzy entropy, the greater the uncertainty of the characteristic parameter relative to the emotion. Combined with the average fuzzy entropy of different emotions, the weight of a certain characteristic parameter is defined as:


(5) $$W_{j}=\frac{\frac{1}{{\left(H_{jk}\right)}_{av}}}{\overset{C}{\underset{k=1}{\sum }}\frac{1}{H_{jk}}}$$

Here is the weight of the feature parameter. The larger the value, the better the resolution of the parameter for different emotions.

Combined with the evolution law of public opinion communication, the theme and emotion in different stages of the life cycle are cooperatively analyzed to generate the theme emotion evolution diagram. On the one hand, it can reveal the thematic relationship and the corresponding emotional distribution characteristics in the same stage, and help the emergency management department to grasp the thematic characteristics of public opinion in each stage more carefully and guide citizens’ emotions. On the other hand, it can track the evolution trend and law of themes and emotions in different stages, which is helpful for emergency management departments to have a more comprehensive understanding of the development track of emergencies and the focus of public attention and the trend of emotions in different stages. Using the method of upper and lower entropy, we can count the word frequency of the online corpus and set the threshold of upper and lower entropy of words. Although most of the words processed by this method are expected network terms, there are still some high-frequency words of non-target words. Rank the importance of words in the corpus, as shown in the formula:


(6) $$TF-IDF\left(w_{i}\right)=\mathrm{freq}\left(w_{i}\right)\cdot \log\left(\frac{N}{df\left(w_{i}\right)}\right)$$

Calculate the importance of each word separately, compare the value with the set limit value, and add words higher than the limit value to the online language emotion dictionary. Determine the polarity intensity of these words, and the calculation formula is as follows:


(7) $$PMI\left(x,y\right)=\log\frac{p\left(x,y\right)}{p\left(x\right)p\left(y\right)}$$

$p\left(x,y\right)$ represents the probability that x and y appear together, $p\left(x\right)$ represents the probability that the word x is used in the text, and $p\left(y\right)$ represents the probability that the word y is used in the text. $PMI\left(x,y\right)$ represents the degree to which two words appear at the same time. Introduce sentiment judgment:


(8) $$\mathrm{SO}\left(w\right)=PMI\left(w,w^{+}\right)-PMI\left(w,w^{-}\right)$$

Where w is the word whose emotional polarity is to be determined. $w^{+}$ and $w^{-}$ represent positive and negative seed words, respectively. If the SO value is greater than the threshold, it means that the word is closer to the word with positive polarity, and the polarity of the word is judged to be positive, otherwise, it is judged to be negative. The output polar words are used as the emotional dictionary of network terms constructed in this paper. To ensure the accuracy of the dictionary, it is necessary to manually label a small number of neutral network words.

## Empirical Analysis of Emotion Dictionary

To verify the effectiveness of the emotion dictionary, it is necessary to use appropriate indicators to evaluate the dictionary. After analyzing the advantages and disadvantages of several evaluation performances, it is decided to adopt two evaluation criteria: accuracy rate and recall rate. Such as formula:


(9) $$r=\frac{n_{1}}{n_{1}+n_{2}}\times 100\%$$

(10) $$p=\frac{n_{1}}{n_{1}+n_{3}}\times 100\%$$

In the formula, *n*_1_ represents the number of words that correctly judged the emotional polarity, that is, the number of words that are consistent with the dictionary and the artificial polarity. *n*_2_ represents the number of words whose emotional polarity judged by the dictionary is inconsistent with the result of manual labeling. *n*_3_ represents the number of words that are not in the search range but are searched by the dictionary. Putting the experimental results into the above formula, respectively, the accuracy and recall rate of the dictionary can be calculated.

**Figure 8:**
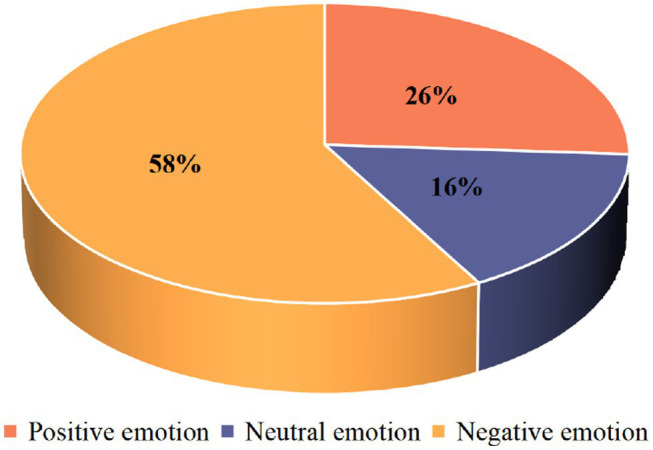
Sentiment analysis of online comments on public opinion events.

After judging the feasibility of the dictionary, the dictionary was used to perform sentiment analysis on 55,303 online comments on the “8.12 Tianjin Big Bang” incident, as shown in Figure 8.

Through the sentiment analysis of the text in the comment section of this topic, it can be seen that positive sentiments accounted for 26% of the total, neutral sentiments accounted for 16% of the total number of comments, and negative sentiment accounted for 58% of the total number of comments. The overall sentiment is in a severely negative state, which is a relatively serious fire-fighting public opinion incident. At this time, relevant government departments should immediately adopt a series of public opinion control measures to adjust the overall emotional trend.

The accuracy (P) and recall (R) are used to evaluate the performance of the emotion dictionary constructed in this paper. The calculated results are shown in Table 4. Only when the domain sentiment dictionary constructed in this paper is better than HowNet sentiment analysis words set and “NTUSD” in accuracy (P) and recall (R) can the sentiment dictionary meet the requirements of network public opinion analysis of public emergencies.

**Table 4:** Evaluation of the performance of each dictionary sentiment classification effect.

	Accuracy (P)	Recall (R)
HowNet emotional analysis words set	0.68	0.73
NTUSD	0.55	0.65
Emotional dictionary of this article	0.86	0.92

The emotion dictionary constructed in this paper has an accuracy rate of 0.86 and a recall rate of 0.92. Through the verification of accuracy and recall rate, the construction method proposed in this paper has good accuracy and reliability. This method of building emotional dictionaries can also be applied to the building of emotional dictionaries in other fields.

## Conclusion

The frequency and scope of emergencies continue to increase, having different impacts on different classes of Chinese society. Crisis communication in the new media environment has its advantages of “first time,” “multi-source,” “people-oriented,” and “decentralization.” There are also problems with the generalization and reinforcement of rumors. The coexistence of advantages and disadvantages of new media also determines the irreplaceable status of traditional media. This paper uses large-scale public emergencies papers, combined with general sentiment dictionaries and deep learning methods to identify the types and strengths of domain sentiment words and sentiment words, and aims to construct a domain sentiment dictionary for public opinion analysis of public emergencies. The emotion dictionary constructed in this paper has an accuracy rate of 0.86 and a recall rate of 0.92. Through the verification of accuracy and recall rate, the construction method proposed in this paper has good accuracy and reliability. After performing sentiment analysis on a specific public opinion event, it can more accurately obtain the emotional state and overall emotional trend of netizens regarding the event, which provides a certain reference value for the government to adopt emergency public opinion management and control methods. In addition, giving full play to the regulating role of emotional distance can effectively control the transformation of communication intentions into specific communication behaviors. According to the user’s friend relationship and emotional trajectory on social media, use social network analysis methods to integrate information, such as communication networks, relationship networks, and interest networks, and establish a public opinion monitoring system by identifying opinion leaders, so as to achieve the purpose of blocking the spread of rumors. Due to the uncertainty of the emotional expression of the text, the artificial judgment of the text emotion will inevitably be biased. There are various types of public emergencies, and different events will have different emotional expression characteristics. Subsequent research needs to further consider the identification of emotional expression characteristics of specific events.

## Data Availability Statement

The original contributions presented in the study are included in the article/supplementary material; further inquiries can be directed to the corresponding author.

## Author Contributions

The author confirms being the sole contributor of this work and has approved it for publication.

## Conflict of Interest

The author declares that the research was conducted in the absence of any commercial or financial relationships that could be construed as a potential conflict of interest.

## Publisher’s Note

All claims expressed in this article are solely those of the authors and do not necessarily represent those of their affiliated organizations, or those of the publisher, the editors and the reviewers. Any product that may be evaluated in this article, or claim that may be made by its manufacturer, is not guaranteed or endorsed by the publisher.

## Abbreviations

CB: Communication behavior
SWC: Spread the will from the crowd
SW: Self-propagating willingness
ALT: Altruism
SLI: Self-interest
POL: Perceptual opinion leader
IE: Immersion experience
AVE: Average variance extracted
CR: Composite reliability.
